# NR4A Family Genes: A Review of Comprehensive Prognostic and Gene Expression Profile Analysis in Breast Cancer

**DOI:** 10.3389/fonc.2022.777824

**Published:** 2022-04-25

**Authors:** Hassan Yousefi, Jordyn Fong, Suresh K. Alahari

**Affiliations:** ^1^ Department of Biochemistry and Molecular Biology, Louisiana State University Health Science Center (LSUHSC), New Orleans, LA, United States; ^2^ Stanley S. Scott Cancer Research Center, Louisiana State University Health Sciences Center, New Orleans, LA, United States; ^3^ Biological Sciences, Louisiana State University, Baton Rouge, LA, United States

**Keywords:** NR4A, breast cancer, survival, TCGA, METABRIC

## Abstract

This report analyzes nuclear receptor (NR) subfamily 4A’s potential role in treating those diagnosed with breast cancer. Here we reviewed the current literature on NR4 family members. We also examined the relative gene expression of the NR4A receptor subfamily in the basal, HER2 (human epidermal growth factor receptor 2) positive, luminal A, and luminal B subtypes using data from tumor samples in The Cancer Genome Atlas (TCGA) and Molecular Taxonomy of Breast Cancer International Consortium (METABRIC). These data showed a positive link between NR4A1-NR4A3 expression and increased overall survival and relapse-free survival in breast cancer patients. In addition, we observed that high expression of NR4A1, NR4A2, and NR4A3 led to better survival. Furthermore, NR4A family genes seem to play an essential regulatory role in glycolysis and oxidative phosphorylation in breast cancer. The novel prognostic role of the NR4A1–NR4A3 receptors implicates these receptors as important mediators controlling breast cancer metabolic reprograming and its progression. The review establishes a strong clinical basis for the investigation of the cellular, molecular, and physiological roles of NR4A genes in breast cancer.

## Introduction

Breast Cancer (BC) is the most common cancer among women and is the second leading cause of cancer deaths in women. Breast cancer affects women worldwide. In 2021, the United States estimated over 280,000 new cases of the invasive disease and over 40,000 deaths (1, 2). This is primarily due to the high metastasis rates, recurrence, and chemoresistance. BC is a heterogeneous and complex disease exhibiting a great degree of intra- and inter-tumoral heterogeneity (3). BC can be categorized into four subtypes: Basal, Human Epidermal growth factor Receptor 2 (HER2) positive, Luminal A, and Luminal B. Each is determined by different genetic or epigenetic factors (4). By such a classification, significant differences have been defined by their risk factors, incidence, treatment sensitivity, and prognosis as the basis for diagnosing and treating this disease (5). Basal, commonly known as Triple Negative Breast Cancer (TNBC), is the most aggressive and malignant subtype since it lacks estrogen receptors (ER), progesterone receptors (PR), and does not express the human epidermal growth factor receptor 2 (HER2) (6). Current traditional treatment options are not as effective at combating this specific breast cancer due to its characteristics. These characteristics make it difficult to treat this subtype with hormone therapies and HER2 inhibitors. Standard treatment options typically involve hormonal therapies and HER2 inhibitors, valid treatment options with other BC subtypes. While therapeutic hormonal therapies for other subtypes are effective, therapeutic resistance can form from prolonged treatment (7). Accordingly, new therapeutic treatments that target specific genes responsible for the regulation and metastasis of tumors are needed to improve the prognosis of patients diagnosed with BC.

This study will be focusing on NR4A (nuclear receptor subfamily 4A), an orphan member of the steroid/thyroid/retinoid nuclear receptor superfamily. These nuclear receptors act as transcription factors to control downstream gene expression and participate in diverse biological functions (8, 9). NR4A receptors are modified by various cell-signaling pathways depending on subcellular localization, levels of expression, transcriptional modulation by coactivators and/or corepressors, posttranslational modification, and interaction with other transcription factors (9). Modulating the expression levels of NR4A1, NR4A2, and NR4A3 can be a potential therapeutic approach for breast cancer. As we outline herein, elucidating the novel prognostic and regulatory roles of the NR4A family and their function as essential mediators regulating breast cancer progression.

## General Functions of NR4A Genes

The homologous orphan receptors, NR4A1, NR4A2, and NR4A3, are a subfamily of the eukaryotic transcription factors (10, 11). This nuclear receptor subfamily encodes 48 human genes and has many biological functions. They have physiological and pathological roles in the human body specifically regulating homeostasis, proliferation, cell migration, apoptosis, metabolism, DNA repair, glucose utilization, and tumorigenesis (12–14). No endogenous ligand has been identified for these nuclear receptors, but they are considered constitutively active (11, 15). Although ligands are not identified, some compounds characterized as ligands that bind the receptor (8, 15). One compound, Cytosporone B (CnsB), an NR4A agonist, has been shown to have increased pro-inflammatory mediators when compared to a control that did not have CnsB (15, 16). NR4A1-3 is commonly considered a molecular switch, and its actions are regulated by a complex network of cellular signaling pathways (11). They are an immediate early gene of stressors and stimulated by peptide hormone, growth factors, cytokines, inflammation, and most importantly, cellular stress (8, 15). They contain a potent sensor that detects changes in the cellular microenvironment, and the NR4A receptors trigger mitochondria biogenesis and improve mitochondrial functions (12, 17). Previous studies have stated that the receptors played roles in promoting DNA repair after cellular stress and can even stimulate protective cells that would aid in the prevention of further damage to cells (14). The same response is also seen in neurons (18). When neurons are exposed to oxidative stress, NR4A is induced through cAMP response element-binding proteins (CREB), upregulated neuroprotective genes, and increased neuronal survival (18). These nuclear receptors also have an important role in immune function and are linked to chronic inflammation, altered immune cell response, and cancer development (8, 11, 12, 19, 20). NR4A3 was identified as a direct transcriptional target of p53 (13). Once p53 is bound to NR4A3, it induces transcription and leads to an increase in expression. This overexpression has been determined to attenuate the proliferation of cancer cells and promote apoptosis through the increasing pro-apoptotic genes (13, 21). The role of the nuclear receptors has been mainly studied in two different cancers: blood-derived cancers and solid tumor cancers (15). The studies on blood-derived cancers have suggested that NR4A has tumor suppressor activity (15). In acute myeloid leukemia (AML), NR4A is silenced by blocking the transcriptional elongation rather than epigenetic promoter silencing (15, 22, 23). Treatment with dihydroergotamine was shown to reactivate NR4A expression and enable elongation of the NR4A promoter, previously hindered by RNA polymerase II (22, 23), and this led to increased survival. Similar trends were shown for Lymphomas, where NR4A3 expression was low and had poor survival (14, 15, 21). Therefore, with an ectopic expression and pharmacological activation, NR4A3 expression increased and was measured to have a higher proportion of apoptotic cells than before the treatment (21). In contrast, solid tumors showed higher levels of NR4A1 and NR4A2 and were exhibited to be prooncogenic (15). An experiment was performed and showed that chimeric antigen receptor T- cells (CAR T-cells) that lacked NR4A promoted tumor regression and prolonged survival of cancer patients when compared to CAR T-cells that expressed NR4A (24). It was determined that NR4A played a role in the cell-intrinsic program of t-cell hyperresponsiveness. Studies that have been performed concluded that an NR4A inducer would be helpful in blood-derived tumors since it would increase the pro-apoptotic genes. At the same time, N4A1/NR4A2 antagonists would benefit solid tumors (15).

## NR4A and Metabolism

Metabolic reprogramming is a typical hallmark of cancer cells (25). Despite adequate oxygen availability, tumor cells tend to generate aerobic glycolysis energy rather than consume the energy produced *via* mitochondrial oxidative phosphorylation (OXPHOS). This phenomenon is known as The Warburg Effect, which often results in increased glucose uptake, an accumulation of ATP, and lactate production in tumor cells in breast cancer, both elevated and reduced OXPHOS activity are observed. In the case of elevated OXPHOS, invasive metastatic breast cancer cells specifically favor mitochondrial respiration to increase ATP levels through a mechanism that involves overexpression of PGC-1α and increases mitochondrial biogenesis (26). It has been shown that OXPHOS activity increases concomitantly with the metastatic potential in primary breast cancer (27, 28). Consistent with this, Alpha CUB-domain containing protein 1 (CDCP1), a transmembrane glycoprotein, drives breast cancer cell metastasis by activating OXPHOS and fatty acid oxidation (26, 29). Therefore, mitochondrial metabolism represents an attractive target for anti-metastatic approaches.

The NR4A subfamily regulates cellular proliferation in a tissue-dependent manner in breast cancer, which means their roles are different in each subtype (19, 30). As described above, in some cancers, the NR4A subfamily can promote proliferation, but in others, they inhibit it. However, results about the roles of the receptors are not consistent. This could result from the nuclear receptors binding lipophilic molecules, which allows it to sense environmental, systemic, and local factors (31). Nuclear receptors derive their ligands from dietary derived factors and metabolism, allowing them to regulate glycolysis and oxidative phosphorylation (9). Previous studies showed an increase in glucagon, which led to an increase in NR4A1, NR4A2, and NR4A3 in a cAMP-dependent process (32). The overexpression of the nuclear receptors induces the expression of other target genes, including Glut2, leading to an increase in gluconeogenesis. This occurs since the expression of the receptors transcriptionally upregulates the rate-limiting enzymes in gluconeogenesis (33). This causes a shift that favors gluconeogenesis, leading to a suppression of glycolysis and, in conjugation, low ATP and cell arrest. In HCC, it has been shown that NR4A1 interacts with phosphoenolpyruvate carboxykinase (PEPCK1), the rate-limiting enzyme in gluconeogenesis, to increase gluconeogenesis and suppress glycolysis, resulting in ATP depletion and cell growth arrest (33). In addition, reduced expression of NR4A1 activated glycolytic pathway in acute promyelocytic leukemia cells (34).

As for the roles of NR4A receptors in the mitochondria, there have been direct links that prove the role of NR4A in mitochondrial production and fuel oxidation. The nuclear receptors are essential for the biogenesis and enzymatic components of the TCA cycle and the ETC (35). Through a downregulation of isocitrate dehydrogenase 1, NR4A1 had been shown to induce oxidative and endoplasmic reticulum stress (36). NR4A1 binds to the mitochondrial bcl-2. In doing so, it weakens the mitochondrial membrane potential. This causes a pro-apoptotic complex (36). These are some mechanisms in which the nuclear receptors can be viewed as having tumor suppression qualities (12). However, there is still a lack of comprehensive bioinformatic study on this topic. Altogether, NR4A receptors are important factors in the processes of carcinogenesis, apoptosis, DNA repair, proliferation, migration, inflammation, metabolism, and angiogenesis (9). Therefore, modulating the expression levels, activity, and nuclear export of NR4A receptors, can develop new cancer treatments. As we outline herein, the novel prognostic role of the NR4A1–NR4A3 receptors implicate these receptors as important mediators controlling breast cancer metabolic reprograming and its progression.

## Bioinformatic Analysis

### NR4A Family Gene Expression Is Associated With the Clinical Outcome of Breast Cancer Patients

**Figure 1:**
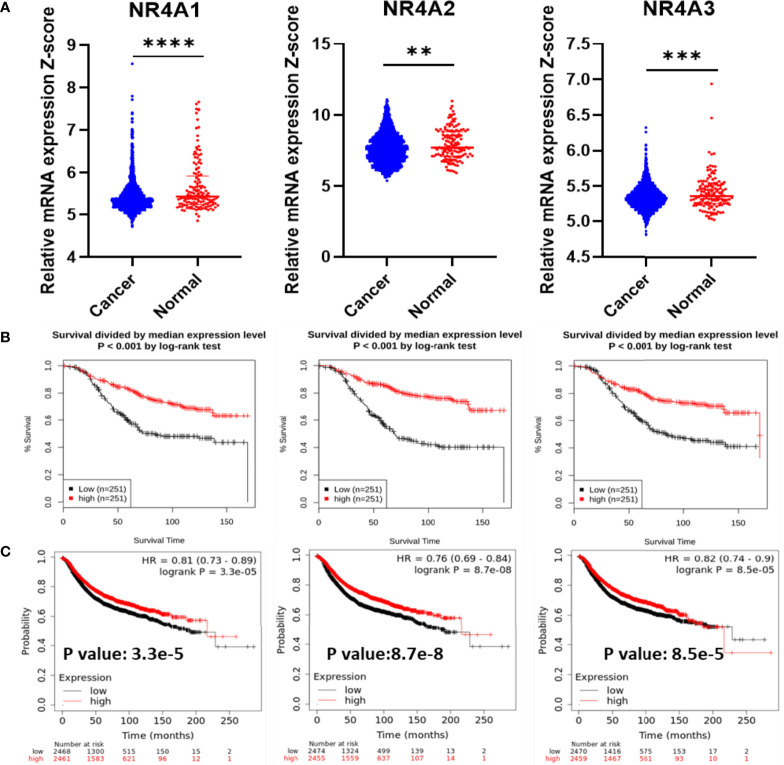
NR4A family gene expression in breast cancer increases survival. **(A)** RNA-Seq mRNA expression data for normal and breast cancer tissue from METABRIC database in normal (n = 148) and cancerous (n = 1826) breast tissue samples. **(B)** Kaplan–Meier overall survival (OS) curves for patients with breast cancer divided by the median value into low and high NR4A mRNA expression (n = 251 per group) using the GENT2 data set. **(C)** Kaplan-Meier analysis (kmplot.com) of relapse-free survival (RFS) based on the mean value of NR4A1, NR4A2, and NR4A3 in breast cancer (n = 4934), with the cutoff values of 282, 373, and 74, respectively. Probes 202340_x_at, 216248_s_at, and 207978_s_at used, respectively. P values calculated using a logrank test for OS and Cox regression model was used for RFS. Data were analyzed by an unpaired t-test. Statistically significant values of **p < 0.01, ***p < 0.001 and ****p < 0.0001 were determined.

First, the mRNA expression of the NR4A receptors was measured in cancer tissues and compared to their mRNA expression in normal tissues (**Figure 1A**). All three receptors showed a significantly lower level of gene expression in cancer tissues when compared to normal tissues (p-value <0.0001, 0.0041, 0.0003, respectively). The GENT2 database was used to graph the overall survival (OS) of breast cancer patients (**Figure 1B**) (37). The expression of the NR4A receptors is divided by the median expression level. In all three receptors, NR4A1, NR4A2, and NR4A3, a higher gene expression level led to better survival (p-value <0.001). We also found that the expression of NR4A receptors was significantly associated with the relapse-free survival (RFS) of breast cancer patients using Kaplan-Meier analysis from (kmplot.com) source (**Figure 1C**) (38). Unfortunately, these datasets only have the median analysis, and thus we could not divide samples into quartile.

### NR4A1, 2 Genes Are Overexpressed in Luminal Breast Cancer While Their Expression Is Downregulated in Basal Breast Cancer

**Figure 2:**
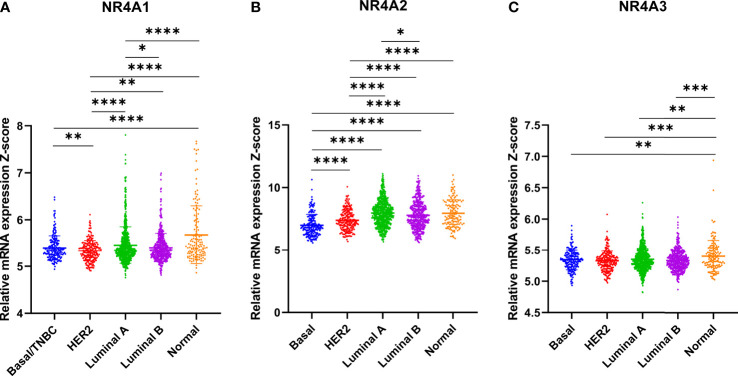
Differential NR4A family gene expression in breast cancer subtypes. **(A–C)** Gene expression of the NR4A1–NR4A3 in METABRIC by Pam50 gene expression subtype classification. Scatterplots show that there is significant association between breast cancer subtypes and the level of NR4A gene expression in breast cancer patients. Basal (n=199), HER2+ (n=220), Lum A (n=679), Lum B (n=461) and Normal-like (n=140) subtypes. Data were analyzed by one-way ANOVA followed by Tukey’s post hoc test. Statistically significant values of *p < 0.05, **p < 0.01, ***p < 0.001 and ****p < 0.0001 were determined.

Using data extracted from METABRIC, the breast cancer subtypes and the expression levels of the receptors were analyzed. NR4A1 showed a significantly lower expression in the basal when compared to the HER2 subtypes, but no other significance was noted in other subtypes (p-value 0.0074) (**Figure 2A**). NR4A2 is significantly under-expressed in the basal subtype relative to all other subtypes (p-value <0.0001) (**Figure 2B**). On the other hand, there was no significant difference in the expression levels of NR4A3 in the basal subtype compared to all other breast cancer subtypes. However, it was down-regulated in all cancerous subtypes compared to the normal subtype (p-value 0.0048, 0.002, 0.0016, and 0.001, respectively) (**Figure 2C**).

### NR4A2 Expression Was Significantly Associated With ER, PR, and HER2 Status

**Figure 3:**
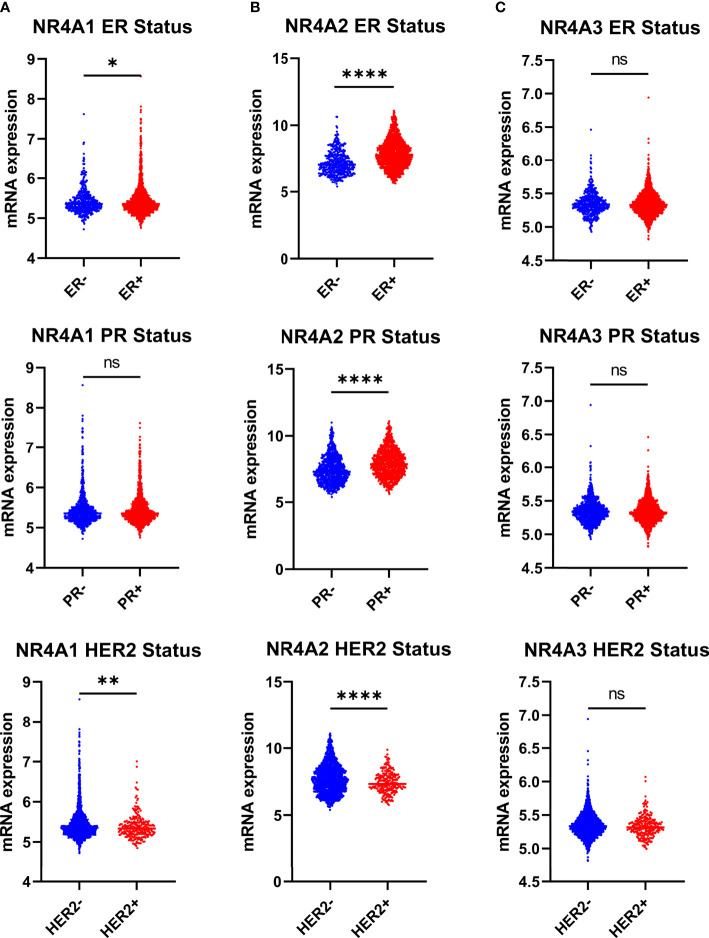
NR4A family gene expression is partially correlating with estrogen receptor breast cancer ER+, PR+ and HER2 subtypes. **(A–C)** Relative expression of NR4A family genes in different breast cancer tumors based on ER (ER+ = 1511, ER- = 474), PR (PR+ = 1040, PR- = 940), and HER2 (HER2+ =247, HER2- = 1733) status examined by IHC. Data were analyzed by an unpaired t-test. Statistically significant values of *p < 0.05, **p < 0.01, ****p < 0.0001, and ns: not significant were determined.

NR4A1 showed significantly lower expression levels in the estrogen receptor-negative and HER2 positive compared to patients’ estrogen receptor-positive and HER2 negative. Still, there was no difference in the progesterone status of BC patients (p-value 0.0407 and 0.0041 respectively) (**Figure 3A**). NR4A2 levels were significantly lower in patients who were ER-, PR-, and HER2+ when compared to their counterparts (**Figure 3B**), while NR4A3 showed no difference (p-value <0.0001, <0.0001, and <0.0001, respectively), (**Figure 3C**).

## Comparision of NR4A Expression With the Stages, Grades, Size, Lymph Node Status of the Tumors, and Age of the Patients

**Figure 4:**
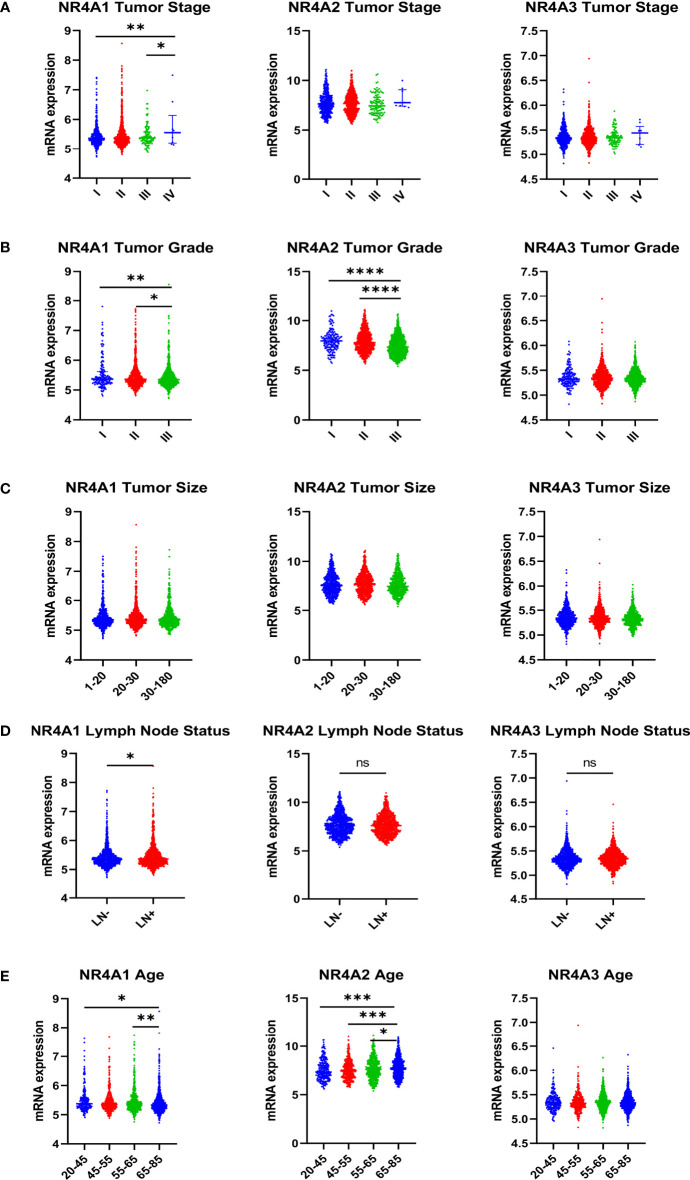
Relative expression of NR4A family genes in different breast cancer tumors, **(A)** stages, **(B)** grades, **(C)** lymph node metastasis, and **(D)** tumor sizes and patients’ age **(E)**. NR4A2 mRNA expression was significantly elevated with increasing age in breast cancer patient while NR4A1 mRNA expression was significantly downregulated with increasing age in breast cancer patient. Tumor size (1-20 = 593, 20-30 = 724, 30-180 =567), tumor stage (I = 475, II = 800, III = 115, IV = 9), tumor grade (I = 165, II = 740, III = 927), lymph node status (LN- = 993, LN+ = 911), Age ( 20-45 = 247, 45-55 = 368, 55-65 = 502, 65-85 = 749, >85 = 38). Data were analyzed by one-way ANOVA followed by Tukey’s post hoc test. Statistically significant values of *p < 0.05, **p < 0.01, ***p < 0.001, ****p < 0.0001, and ns, not significant were determined.

A significantly higher level of NR4A1 expression was found in tumor stage four relative to tumor stage one and three. However, there was no difference in the levels of the other receptors (p-value 0.0099 and 0.0218, respectively), (**Figure 4A**). NR4A1 and NR4A2 had significantly lower expression in grade 3 tumors when compared to grades 1 and 2, but there was no difference when evaluating the expression of NR4A3 (p-value 0.0011, 0.0338, <0.0001, and <0.0001, respectively) (**Figure 4B**). All three nuclear receptors’ expression levels did not influence tumor size (**Figure 4C**). NR4A1 showed a significantly higher gene expression in patients that were diagnosed lymph node-positive relative to those lymph node-negative. At the same time, there was no significant difference in the levels for NR4A2 and NR4A3 (p-value 0.0308) (**Figure 4D**). NR4A1 showed a significantly lower gene expression in those 65-85 when compared to those 20-45 and 55-65 (p-value 0.0167 and 0.0046, respectively). However, the opposite trend was shown for NR4A2, with those 65-85 having a significantly higher gene expression than all other groups (p-value 0.0003, 0.0001, 0.0453, respectively). Finally, there was no significant difference in NR4A3 expression between age groups (**Figure 4E**).

### NR4A Family DNA Methylation Was Not Associated With Gene Expression in Breast Cancer

**Figure 5:**
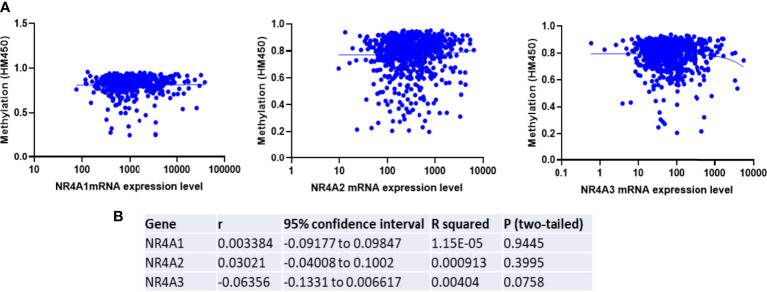
Expression of NR4A family gene is not correlated with the promoter methylation in breast tumors. **(A)** TCGA breast invasive carcinoma DNA methylation (Illumina Infinium HumanMethylation450) and gene expression microarray datasets were analyzed. **(B)** Representative statistics demonstrated correlation between NR4A family gene expression and its DNA methylation. Gene expression were not found to be correlated with DNA methylation.

To determine if epigenetic regulation of the NR4A family gene promoter contributes to the expression of NR4A genes, we analyzed methylation levels of the NR4A1, 2, and 3 genes using the TCGA HumanMethylation450 Array patient data. Correlation analysis in the TCGA dataset showed a non-significant correlation between methylation and expression levels of NR4A1 and 2 and a non-significant inverse correlation between methylation and expression levels of NR4A3 in breast cancer patients (**Figures 5A, B**). These results indicate that DNA methylation of NR4A genes may not be a potential epigenetic modification resulting in the expression differences between the cancerous and normal-like subtypes of breast cancer.

### NR4As Co-Expression Status With Glycolysis and Oxidative Phosphorylation Gene Hallmarks in Breast Cancer

**Figure 6:**
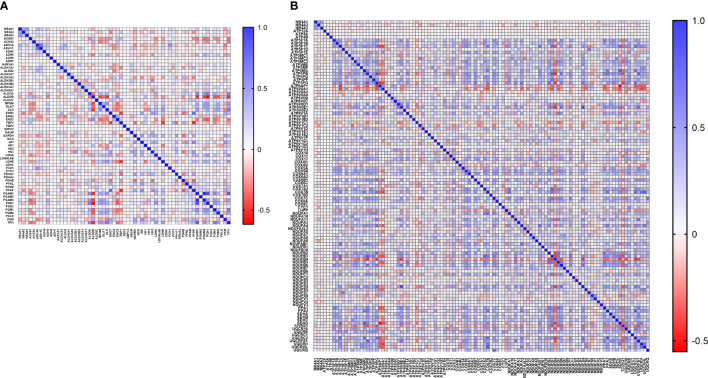
Co-expression correlation analysis between NR4A1–NR4A3 genes and the **(A)** glycolysis and **(B)** oxidative phosphorylation marker genes related markers using HALLMARK_GLYCOLYSIS and KEGG_OXIDATIVE_PHOSPHORYLATION gene sets. The association between genes was measured using the Pearson correlation coefficient (r). Correlation heatmap (Pearson r) of the transcriptomes from METABRIC breast cancer project samples (n= 1985). Red color refers to negative correlation, and the blue color indicates positive correlation.

**Figure 7:**
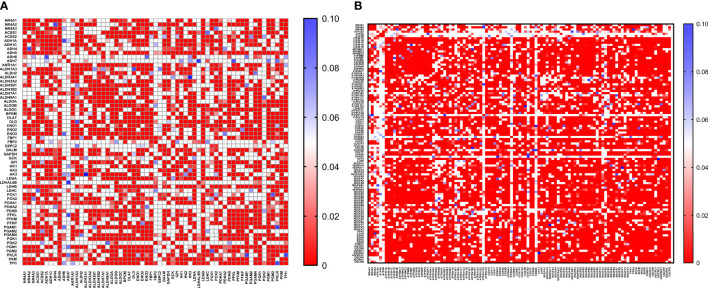
Co-expression correlation analysis between NR4A1–NR4A3 genes and the **(A)** glycolysis and **(B)** oxidative phosphorylation marker genes related markers using HALLMARK_GLYCOLYSIS and KEGG_OXIDATIVE_PHOSPHORYLATION gene sets. The association between genes was measured using the Pearson correlation coefficient (r). Correlation heatmap (P values) of the transcriptomes from METABRIC breast cancer project samples (n= 1985). Red color refers to P values< 0.05, and the white and blue colors indicates P values > 0.05.

With the availability of METABRIC as a big dataset, we performed co-expression correlation analysis between NR4A genes and the glycolysis and oxidative phosphorylation gene sets (Gene Set: HALLMARK_GLYCOLYSIS (39) and Oxidative Phosphorylation (Kegg: 00190)) using the mRNA expression data from 1904 patients. Our data showed that NR4A1 has a negative correlation with 16 genes from the pathway and 26 genes in the oxidative phosphorylation pathway (**Figure 6A, B**). These data were further analyzed incorporating p values (**Figures 7A, B**). In the case of NR4A2, we have seen 26 and 39 genes negatively correlated with NR4A2 expression in glycolysis and oxidative phosphorylation, respectively showing that NR4A2 might have a key role in suppressing these pathways compared to the other NR4A family members. Also, NR4A3 expression was negatively correlated with 9 and 30 genes in glycolysis and oxidative phosphorylation pathways, respectively. Altogether, this result shows NR4A family genes might play an important regulatory role in glycolysis and oxidative phosphorylation in breast cancer.

## Methods

### cBioPortal Database Analysis

The PAM50 gene signature was used to analyze BC patients’ different intrinsic molecular subtypes (40). This was achieved using two publicly available databases, the TCGA (41) and the METABRIC (42), containing clinical reports for BC patients. The TCGA project (1108 of Breast Invasive Carcinoma from TCGA (TCGA, Firehose Legacy)) contains gene expression profiles, methylation, copy number variation and mutation information from different cancer types. In addition, METABRIC dataset contain gene expression data and corresponding clinical data of targeted sequencing of 2509 primary breast tumors with 548 matched normals. The RNASeq data was performed on Breast Invasive Carcinoma samples with clinical information available at the TCGA data portal, cBioPortal for Cancer Genomics (http://www.cbioportal.org/). In the case of TCGA, gene expression data is reported as in RSEM normalized count (log intensity levels) for 1108 Breast Invasive Carcinoma samples. Gene expression data from 1904 breast tumors (Expression log intensity levels (Illumina Human v3 microarray)) and clinical information data from METABRIC were also used. According to the PAM50 classification, METABRIC BC dataset was divided into 5 subtypes, including the Basal (n=199), HER2+ (n=220), Lum A (n=679), Lum B (n=461) and Normal-like (n=140) subtypes. Clinical data for each TCGA sample is downloaded directly from the TCGA Data Portal. ER, PR, and, HER2 status are assessed using the IHC information. The expression analyses of NR4A1–NR4A3 transcripts in the subtypes and histological grades were performed using one-way analysis of variance (one-way ANOVA) and the student’s t-test. All analyses are performed in Graphpad Prism, version 8. P-values <0.05 were considered statistically significant differences.

### Survival Analysis

Kaplan–Meier Plotter (http://www.kmplot.com) is an online public database evaluating the effect of genes on patient clinical outcomes in different cancers. This public tool is operated by a PostgreSQL server that could integrate gene expression and clinical data simultaneously The Kaplan-Meier Plotter database (http://kmplot.com/analysis/) (38) was used to analyze the correlation between the expression levels of NR4A1–NR4A3 and breast cancer patient relapse-free survival (RFS). Kaplan-Meier analysis (kmplot.com) of relapse-free survival (RFS) based on the mean value of NR4A1, NR4A2, and NR4A3 in breast cancer (n = 4934), with the cutoff values of 282, 373, and 74, respectively. Probes 202340_x_at, 216248_s_at, and 207978_s_at used, respectively. In addition, Kaplan-Meier overall survival analysis of breast cancer patients divided by the median expression level (into low and high NR4A mRNA expression (n = 251 per group) of the NR4A1–NR4A3 family was performed in a platform for exploring Gene Expression patterns across Normal and Tumor tissues named GENT2 (http://gent2.appex.kr/gent2/) (37). P values calculated using a log-rank test for OS and Cox regression model was used for RFS. Also, a data was analyzed by one-way ANOVA followed by t-test.

### Correlation Analysis

The OXPHOS-related gene signature comprised 132 genes obtained from the gene set “KEGG_OXIDATIVE_PHOSPHORYLATION” in The Molecular Signatures Database hallmark gene sets (MsigDB, software.broadinstitute.org/gsea/msigdb). The Glycolysis-related gene signature comprised 200 genes obtained from the gene set “HALLMARK_GLYCOLYSIS” in The Molecular Signatures Database hallmark gene sets. Then, the mRNA expression levels of these genes has been extracted from METABRIC and Pearson’s correlation analysis was used to to test their co-expression. All analyses are performed in GraphPad Prism, version 8.

### Methylation Analysis

DNA methylation (Illumina Infinium HumanMethylation450) datasets were extracted from UCSC Cancer Browser (https://www.cancer.gov/tcga), along with the clinical-pathological phenotypes. Methylation (HM450) beta-values for genes in 885 cases (Breast Invasive Carcinoma (TCGA, Firehose Legacy) were used. The data was downloaded from the TCGA database. The RNA-seq data and methylation data for level 3 were downloaded from TCGA, and the selected samples were all patient tissue samples. In the downloaded RNA-seq data and methylation data, the data with incomplete clinical information were excluded. Only the samples that had undergone RNA sequencing and methylated chip data were retained in the remaining data to make it possible to perform a linkage analysis of transcription and methylation. Pearson’s correlation test calculated the correlation between the gene methylation degree and the corresponding gene expression. As mentioned, Pearson’s correlation analysis was used to analyze of the correlation between the methylation degree and gene expression.

## Summary

According to the 2021 American Cancer Society statistics, BC has the highest number of estimated new cases and the second-highest number of deaths (43). There are multiple available treatments for BC that have proven effective. However, there are often cases of relapse or drug resistance following the completion of treatment. Therefore, it is important to emphasize that BC diagnosis time is critical, as early detection will allow for quicker treatment intervention. The NR4A family of orphan receptors, NR4A1–NR4A3, belongs to the superfamily of nuclear receptors, which regulate genes involved in proliferation, cell migration, and apoptosis. Here, we report on the clinical and prognostic value of NR4As and their relevance in prognosis and therapeutics for BC patients. Using the large genomic studies, TCGA and METABRIC, we were able to analyze the expression of NR4A1–NR4A3 in all BC subtypes, including Basal, Her2, Luminal A, and Luminal B. Basal BC is considered to be aggressive and metastatic, and effective therapeutic treatments are minimal (44). NR4A1 and NR4A2 have been studied in breast cancer (30, 45, 46). Consistent with our analysis, it has been shown that NR4A1 reduced during the development of mouse basal-like mammary tumors and significantly downregulated in human TNBC samples. More specifically, TNBC cell lines with little endogenous NR4A1 inhibited cell proliferation, viability, migration, and invasion by down-regulating the JNK1–AP-1–cyclin D1 pathway (45). This is probably why this subtype has a very poor prognosis and is often associated with cancer relapse following completion of treatment. Also, the nuclear receptor NR4A2 participates in multiple metabolic regulations and plays paradoxical roles in tumorigeneses (11). For example, NR4A2 interacted and recruited corepressors, the SWI/SNF complex, to the promoters of CD36 and FABP4 to suppress their transcriptions. Because of this, the fatty acid uptake was hampered, leading to the inhibition of breast cancer cell proliferation (46). In another study, Llopis, S., et al. showed the dichotomous roles of the NR4A2 in breast cancer (30). They showed a higher NR4A2 expression level in the normal breast epithelium compared to breast carcinoma cells, which strongly correlated with an increase in relapse-free survival in a cohort of breast cancer patients. However, NR4A2-silenced breast xenografts models showed significantly decreased growth compared to the control model, showing a biphasic role for NR4A2 in breast cancer progression (30). NR4A3 has been previously shown to be a direct target for p53, inducing its transcription. Mechanistically, overexpression of NR4A3 attenuated the proliferation of breast cancer cells and promoted apoptosis by augmenting the expression of pro-apoptotic genes PUMA and Bax (13).

To date, there is no patient-centered clinical dataset validating the role of NR4A gene expression in breast cancer. Thus, we sought to investigate the role and functionality of NR4A in BC patients using publicly available databases containing records from large BC studies. Multiple research groups have extensively studied the role and therapeutic benefits of NR4As as a potent tumor suppressor or oncogene protein in breast cancer and, more specifically, how it depends on the tumor subtypes. In this study, we characterized gene expression of NR4A1–NR4A3 in all the breast cancer subtypes (Basal, Her2, Luminal A, and Luminal B) by using large genomic studies (TCGA and METABRIC). Previous studies found NR4A family gene expression critical in basal breast cancer progression and metastasis (30, 35, 36, 45). Our study found that NR4A1, 2, and 3 gene expressions are luminal breast cancer-specific. Low gene expression of NR4A1 is observed in basal, HER2, and luminal A subtypes compared to the Normal-like subtype. NR4A2 mRNA overexpressed in both luminal A and luminal B breast cancer relative to other subtypes. NR4A3 mRNA expression was lower in all cancer subtypes compared to Normal-like subtypes with no meaningful differential expression between cancerous subtypes. In addition, NR4A1 and NR4A2 mRNA expressions are lower in basal breast cancer when compared to other subtypes of breast cancer. Also, when samples from all BC intrinsic subtypes were analyzed as a group, NR4A1-NR4A3 expression is significantly downregulated in BC patients compared to normal control subjects. Previously, using publicly available clinical data and patient survival analysis has been shown that having high levels of NR4A3 expression positively correlates with increased survival rates for patients with breast cancer (13). Consistent with this, our results showed a positive link between NR4A1-NR4A3 expression and increased overall survival and relapse-free survival in breast cancer patients.

Interestingly, we found that NR4A1 expression is progressively downregulated with an increase in age while NR4A2 is upregulated. Previous studies have shown in HCC it has been demonstrated that NR4A1 suppresses glycolysis in different cancers (33, 34). The result of our correlation analysis validated that NR4A1-NR4A3 expression is reversely correlated with many glycolytic and oxidative phosphorylation targets, suggesting the probable role of these receptors in regulating these pathways in breast cancer. Finally, our methylation results showed no significant positive correlation between NR4A1-NR4A3 expression and their DNA methylation, suggesting that these genes’ epigenetic regulation might not be the case in breast cancer. In summary this review indicates prognostic role of the NR4A1–NR4A3 receptors and implicates these receptors as important mediators controlling breast cancer metabolic reprograming and its progression.

## Data Availability Statement

The raw data supporting the conclusions of this article will be made available by the authors, without undue reservation.

## Author Contributions

HY and JF wrote the first draft and SA finalized the manuscript. All authors contributed to the article and approved the submitted version.

## Conflict of Interest

The authors declare that the research was conducted in the absence of any commercial or financial relationships that could be construed as a potential conflict of interest.

## Publisher’s Note

All claims expressed in this article are solely those of the authors and do not necessarily represent those of their affiliated organizations, or those of the publisher, the editors and the reviewers. Any product that may be evaluated in this article, or claim that may be made by its manufacturer, is not guaranteed or endorsed by the publisher.

## References

[1] Sung H Ferlay J Siegel RL Laversanne M Soerjomataram I Jemal A . Global Cancer Statistics 2020: GLOBOCAN Estimates of Incidence and Mortality Worldwide for 36 Cancers in 185 Countries. CA Cancer J Clin (2021) 71(3):209–49. doi: 10.3322/caac.21660 33538338

[2] Waks AG Winer EP . Breast Cancer Treatment: A Review. Jama (2019) 321(3):288–300. doi: 10.1001/jama.2018.19323 30667505

[3] Marusyk A Almendro V Polyak K . Intra-Tumour Heterogeneity: A Looking Glass for Cancer? Nat Rev Cancer (2012) 12(5):323–34. doi: 10.1038/nrc3261 22513401

[4] Saha I Rakshit S Wlasnowolski M Plewczyński D . Identification of Epigenetic Biomarkers With the Use of Gene Expression and DNA Methylation for Breast Cancer Subtypes. In: Tencon 2019-2019 Ieee Region 10 Conference (Tencon). Kochi, India: IEEE (2019).

[5] Gu G Dustin D Fuqua SA . Targeted Therapy for Breast Cancer and Molecular Mechanisms of Resistance to Treatment. Curr Opin Pharmacol (2016) 31:97–103. doi: 10.1016/j.coph.2016.11.005 27883943

[6] Fertig EJ Lee E Pandey NB Popel AS . Analysis of Gene Expression of Secreted Factors Associated With Breast Cancer Metastases in Breast Cancer Subtypes. Sci Rep (2015) 5(1):1–11. doi: 10.1038/srep12133 PMC4648401 26173622

[7] Rexer BN Arteaga CL . Intrinsic and Acquired Resistance to HER2-Targeted Therapies in HER2 Gene-Amplified Breast Cancer: Mechanisms and Clinical Implications. Crit Rev Oncogen (2012) 17(1):1–16. doi: 10.1615/CritRevOncog.v17.i1.20 PMC3394454 22471661

[8] Safe S Jin UH Morpurgo B Abudayyeh A Singh M Tjalkens RB . Nuclear Receptor 4a (NR4A) Family - Orphans No More. J Steroid Biochem Mol Biol (2016) 157:48–60. doi: 10.1016/j.jsbmb.2015.04.016 25917081 PMC4618773

[9] Mohan HM Aherne CM Rogers AC Baird AW Winter DC Murphy EP . Molecular Pathways: The Role of NR4A Orphan Nuclear Receptors in Cancer. Clin Cancer Res (2012) 18(12):3223–8. doi: 10.1158/1078-0432.CCR-11-2953 22566377

[10] Beard JA Tenga A Chen T . The Interplay of NR4A Receptors and the Oncogene-Tumor Suppressor Networks in Cancer. Cell Signal (2015) 27(2):257–66. doi: 10.1016/j.cellsig.2014.11.009 PMC4276441 25446259

[11] Ranhotra HS . The NR4A Orphan Nuclear Receptors: Mediators in Metabolism and Diseases. J Recept Signal Transduct Res (2015) 35(2):184–8. doi: 10.3109/10799893.2014.948555 25089663

[12] Crean D Murphy EP . Targeting NR4A Nuclear Receptors to Control Stromal Cell Inflammation, Metabolism, Angiogenesis, and Tumorigenesis. Front Cell Dev Biol (2021) 9:589770. doi: 10.3389/fcell.2021.589770 33634114 PMC7901948

[13] Fedorova O Petukhov A Daks A Shuvalov O Leonova T Vasileva E . Orphan Receptor NR4A3 is a Novel Target of P53 That Contributes to Apoptosis. Oncogene (2019) 38(12):2108–22. doi: 10.1038/s41388-018-0566-8 30455429

[14] Wenzl K Troppan K Neumeister P Deutsch AJ . The Nuclear Orphan Receptor NR4A1 and NR4A3 as Tumor Suppressors in Hematologic Neoplasms. Curr Drug Targets (2015) 16(1):38–46. doi: 10.2174/1389450115666141120112818 25410408

[15] Safe S Shrestha R Mohankumar K . Orphan Nuclear Receptor 4a1 (NR4A1) and Novel Ligands. Essays Biochem (2021) 65(6):877–86. doi: 10.1042/EBC20200164 PMC11410023 34096590

[16] Ismaiel M Murphy B Aldhafiri S Giffney HE Thornton K Mukhopadhya A . The NR4A Agonist, Cytosporone B, Attenuates Pro-Inflammatory Mediators in Human Colorectal Cancer Tissue Ex Vivo. Biochem Biophys Res Commun (2021) 554:179–85. doi: 10.1016/j.bbrc.2021.03.110 33798945

[17] Paillasse MR de Medina P . The NR4A Nuclear Receptors as Potential Targets for Anti-Aging Interventions. Med Hypotheses (2015) 84(2):135–40. doi: 10.1016/j.mehy.2014.12.003 25543265

[18] Volakakis N Kadkhodaei B Joodmardi E Wallis K Panman L Silvaggi J . NR4A Orphan Nuclear Receptors as Mediators of CREB-Dependent Neuroprotection. Proc Natl Acad Sci USA (2010) 107(27):12317–22. doi: 10.1073/pnas.1007088107 PMC2901488 20566846

[19] Beard JA Tenga A Chen T . The Interplay of NR4A Receptors and the Oncogene–Tumor Suppressor Networks in Cancer. Cell Signal (2015) 27(2):257–66. doi: 10.1016/j.cellsig.2014.11.009 PMC4276441 25446259

[20] Smith AG Lim W Pearen M Muscat GE Sturm RA . Regulation of NR4A Nuclear Receptor Expression by Oncogenic BRAF in Melanoma Cells. Pigment Cell Melanoma Res (2011) 24(3):551–63. doi: 10.1111/j.1755-148X.2011.00843.x 21362156

[21] Deutsch AJA Rinner B Pichler M Prochazka K Pansy K Bischof M . NR4A3 Suppresses Lymphomagenesis Through Induction of Proapoptotic Genes. Cancer Res (2017) 77(9):2375–86. doi: 10.1158/0008-5472.CAN-16-2320 28249906

[22] Call SG Duren RP Panigrahi AK Nguyen L Freire PR Grimm SL . Targeting Oncogenic Super Enhancers in MYC-Dependent AML Using a Small Molecule Activator of NR4A Nuclear Receptors. Sci Rep (2020) 10(1):2851. doi: 10.1038/s41598-020-59469-3 32071334 PMC7029036

[23] Boudreaux SP Duren RP Call SG Nguyen L Freire PR Narayanan P . Drug Targeting of NR4A Nuclear Receptors for Treatment of Acute Myeloid Leukemia. Leukemia (2019) 33(1):52–63. doi: 10.1038/s41375-018-0174-1 29884904 PMC6286710

[24] Chen J López-Moyado iF Seo H Lio CJ Hempleman LJ Sekiya T . NR4A Transcription Factors Limit CAR T Cell Function in Solid Tumours. Nature (2019) 567(7749):530–4. doi: 10.1038/s41586-019-0985-x PMC6546093 30814732

[25] Pavlova NN Thompson CB . The Emerging Hallmarks of Cancer Metabolism. Cell Metab (2016) 23(1):27–47. doi: 10.1016/j.cmet.2015.12.006 26771115 PMC4715268

[26] LeBleu VS O'Connell JT Gonzalez Herrera KN Wikman H Pantel K Haigis MC . PGC-1α Mediates Mitochondrial Biogenesis and Oxidative Phosphorylation in Cancer Cells to Promote Metastasis. Nat Cell Biol (2014) 16(10):992–1003. doi: 10.1038/ncb3039 25241037 PMC4369153

[27] Lehuédé C Dupuy F Rabinovitch R Jones RG Siegel PM . Metabolic Plasticity as a Determinant of Tumor Growth and Metastasis. Cancer Res (2016) 76(18):5201–8. doi: 10.1158/0008-5472.CAN-16-0266 27587539

[28] Simões RV Serganova IS Kruchevsky N Leftin A Shestov AA Thaler HT . Metabolic Plasticity of Metastatic Breast Cancer Cells: Adaptation to Changes in the Microenvironment. Neoplasia (2015) 17(8):671–84. doi: 10.1016/j.neo.2015.08.005 PMC4674487 26408259

[29] Wright HJ Hou J Xu B Cortez M Potma EO Tromberg BJ . CDCP1 Drives Triple-Negative Breast Cancer Metastasis Through Reduction of Lipid-Droplet Abundance and Stimulation of Fatty Acid Oxidation. Proc Natl Acad Sci (2017) 114(32):E6556–65. doi: 10.1073/pnas.1703791114 PMC5559020 28739932

[30] Llopis S Singleton B Duplessis T Carrier L Rowan B Williams C . Dichotomous Roles for the Orphan Nuclear Receptor NURR1 in Breast Cancer. BMC Cancer (2013) 13:139. doi: 10.1186/1471-2407-13-139 23517088 PMC3617898

[31] Thorne JL Campbell MJ . Nuclear Receptors and the Warburg Effect in Cancer. Int J Cancer (2015) 137(7):1519–27. doi: 10.1002/ijc.29012 PMC4790452 24895240

[32] Chao LC Wroblewski K Ilkayeva OR Stevens RD Bain J Meyer GA . Skeletal Muscle Nur77 Expression Enhances Oxidative Metabolism and Substrate Utilization [s]. J Lipid Res (2012) 53(12):2610–9. doi: 10.1194/jlr.M029355 PMC3494265 23028113

[33] Bian X-l Chen H-Z Yang P-B Li Y-P Zhang F-N Zhang J-Y . Nur77 Suppresses Hepatocellular Carcinoma via Switching Glucose Metabolism Toward Gluconeogenesis Through Attenuating Phosphoenolpyruvate Carboxykinase Sumoylation. Nat Commun (2017) 8(1):1–14. doi: 10.1038/ncomms14420 28240261 PMC5333363

[34] Corrocher FA Bueno de Paiva L Duarte ASS Ferro KP Silveira LDR de Lima TI . Reduced Expression of NR4A1 Activates Glycolytic Pathway in Acute Promyelocytic Leuk Cells. Leuk Lymphoma (2018) 59(6):1501–4. doi: 10.1080/10428194.2017.1387900 29041844

[35] Herring JA Elison WS Tessem JS . Function of Nr4a Orphan Nuclear Receptors in Proliferation, Apoptosis and Fuel Utilization Across Tissues. Cells (2019) 8(11):1373. doi: 10.3390/cells8111373 PMC6912296 31683815

[36] Hedrick E Lee SO Doddapaneni R Singh M Safe S . Nuclear Receptor 4A1 as a Drug Target for Breast Cancer Chemotherapy. Endocr Relat Cancer (2015) 22(5):831–40. doi: 10.1530/ERC-15-0063 26229035

[37] Park S-J Yoon B-H Kim S-K Kim S-Y . GENT2: An Updated Gene Expression Database for Normal and Tumor Tissues. BMC Med Genomics (2019) 12(5):1–8. doi: 10.1186/s12920-019-0514-7 31296229 PMC6624177

[38] Nagy Á. Munkácsy G Győrffy B . Pancancer Survival Analysis of Cancer Hallmark Genes. Sci Rep (2021) 11(1):1–10. doi: 10.1038/s41598-021-84787-5 33723286 PMC7961001

[39] Liberzon A Birger C Thorvaldsdóttir H Ghandi M Mesirov JP Tamayo P . The Molecular Signatures Database Hallmark Gene Set Collection. Cell Syst (2015) 1(6):417–25. doi: 10.1016/j.cels.2015.12.004 PMC4707969 26771021

[40] Cancer Genome Atlas N . Comprehensive Molecular Portraits of Human Breast Tumours. Nature (2012) 490(7418):61–70. doi: 10.1038/nature11412 23000897 PMC3465532

[41] The Cancer Genome Atlas Research Network Weinstein JN Collisson EA Mills GB Mills Shaw KR Ozenberger BA . The Cancer Genome Atlas Pan-Cancer Analysis Project. Nat Genet (2013) 45(10):1113–20. doi: 10.1038/ng.2764 PMC3919969 24071849

[42] Pereira B Chin S-F Rueda OM Moen Vollan H-K Provenzano E Bardwell HA . The Somatic Mutation Profiles of 2,433 Breast Cancers Refine Their Genomic and Transcriptomic Landscapes. Nat Commun (2016) 7(1):1–16. doi: 10.1038/ncomms11479 PMC4866047 27161491

[43] Siegel RL Miller KD Fuchs HE Jemal A . Cancer Statistics, 2021. CA Cancer J Clin (2021) 71(1):7–33. doi: 10.3322/caac.21654 33433946

[44] Alluri P Newman LA . Basal-Like and Triple-Negative Breast Cancers: Searching for Positives Among Many Negatives. Surg Oncol Clin North Am (2014) 23(3):567–77. doi: 10.1016/j.soc.2014.03.003 PMC4304394 24882351

[45] Wu H Bi J Peng Y Huo L Yu X Yang Z . Nuclear Receptor NR4A1 Is a Tumor Suppressor Down-Regulated in Triple-Negative Breast Cancer. Oncotarget (2017) 8(33):54364. doi: 10.18632/oncotarget.17532 28903348 PMC5589587

[46] Yang PB Hou PP Liu FY Hong WB Chen HZ Sun XY . Blocking Pparγ Interaction Facilitates Nur77 Interdiction of Fatty Acid Uptake and Suppresses Breast Cancer Progression. Proc Natl Acad Sci (2020) 117(44):27412–22. doi: 10.1073/pnas.2002997117 PMC7959534 33087562

